# Comparison of Periodontopathic Bacterial Profiles of Different Periodontal Disease Severity Using Multiplex Real-Time Polymerase Chain Reaction

**DOI:** 10.3390/diagnostics10110965

**Published:** 2020-11-17

**Authors:** Jin Uk Choi, Jun-Beom Lee, Kyoung-Hwa Kim, Sungtae Kim, Yang-Jo Seol, Yong-Moo Lee, In-Chul Rhyu

**Affiliations:** 1 Department of Periodontology and Dental Research Institute, School of Dentistry, Seoul National University, Seoul 03080, Korea; choi7767@snu.ac.kr (J.U.C.); khk6911@hanmail.net (K.-H.K.); kst72@snu.ac.kr (S.K.); yjseol@snu.ac.kr (Y.-J.S.); ymlee@snu.ac.kr (Y.-M.L.); 2 Department of Periodontics, Seoul National University Dental Hospital, Seoul 03080, Korea; dentjblee@gmail.com

**Keywords:** multiplex polymerase chain reaction, periodontitis, diagnosis, bacteria, saliva, biomarkers

## Abstract

Periodontopathic bacteria are known to have a pivotal role in the pathogenesis of periodontitis. The aim of the study was to quantitatively compare bacterial profile of patients with different severity of periodontal disease using samples from mouthwash and the subgingival area. Further analysis was performed to evaluate the correlation between mouthwash and two subgingival sampling methods: paperpoint and gingival retraction cord; 114 subjects enrolled in the study, and were divided equally into three groups according to disease severity. Mouthwash and subgingival sampling were conducted, and the samples were quantitatively analyzed for 11 target periodontopathic bacteria using multiplex real-time PCR. There were statistically significant differences in bacterial counts and prevalence of several species between the study groups. Mouthwash sampling showed significant correlations with two different subgingival sampling methods in regard to the detection of several bacteria (e.g., *ρ* = 0.793 for *Porphyromonas gingivalis* in severe periodontitis), implying that mouthwash sampling can reflect subgingival microbiota. However, the correlation was more prominent as disease severity increased. Although bacteria in mouthwash have potential to become a biomarker, it may be more suitable for the diagnosis of severe periodontitis, rather than early diagnosis. Further research is required for the discovery of biomarkers for early diagnosis of periodontitis.

## 1. Introduction

Periodontitis is inflammatory conditions of the tooth-supporting structures, which results in the destruction of periodontium and finally leads to the loss of tooth [1]. Since the inflammation is known to be caused by the host response to oral microbial biofilm [2], the role of oral microbiome in the pathogenesis of the disease has been extensively explored for a long period. Various hypotheses, such as nonspecific plaque hypothesis and specific plaque hypothesis, have been proposed to explain the process of periodontal tissue destruction [3]. More recently, ecological plaque hypothesis was introduced, suggesting that the accumulation of dental plaque around the gingival margin may provide ecological stresses that favor the proliferation of anaerobic Gram-negative bacteria, and finally cause tissue-destructive host response [4]. These ecological shifts in the composition of subgingival microbiota, that is, the proliferation of different Gram-negative anaerobes over facultative Gram-positive species in the periodontal pocket, is well documented to be associated with the development and progression of periodontitis [5].

Socransky and his coworkers [6] suggested a detailed analysis of the microbial complexes in the subgingival plaque. The cluster analysis yielded six closely associated bacterial complexes, and they were designated with color codes. Four complexes mainly consist of early colonizers of the tooth surface, namely, “Blue complex” consisting of *Actinomyces* species, “Yellow complex” consisting of various *Streptococci*, “Green complex” consisting of *Eiknella corrodens* and *Capnocytophaga* species, and “Purple complex” consisting of *Veillonella parvula* and *Actinomyces odontolyticus*. Two additional complexes were recognized. “Orange complex” includes various species of *Prevotella*, *Fusobacterium*, *Campylobacter* and other bacteria. “Red complex” comprises three bacterial species, that is, *Porphyromonas gingivalis*, *Tannerella forsythia*, and *Treponema denticola*. These two complexes have been classified as late colonizer in the development and maturation of subgingival plaque in the periodontal pocket and they have been closely related to the pathological conditions of periodontal tissue. This classification system is still valid in the area of periodontal microbiology.

A variety of sampling techniques have been utilized for the microbiological assessment. To acquire the sample of subgingival microbiota from the periodontal pocket, the insertion of sterile paperpoint or curette into the periodontal pocket were widely utilized. However, they may be invasive and difficult to perform, so a well-trained practitioner is required for sampling procedure [7]. Recently, saliva is considered to be more appropriate for the diagnostic aid in daily practice due to its easy and non-invasive sampling procedure [8]. Interestingly, the salivary microbiota of periodontitis patients was presented to be different from that of healthy subjects. A microarray-based research by Belstrom and coworkers [9] reported eight bacterial taxa, and four bacterial clusters were observed to be present statistically more frequently in samples from periodontitis. Another study conducted by Chen and colleagues [10] demonstrated that six genera including *Porphyromonas*, *Tannerella*, and *Eubacterium* in the saliva sample of periodontitis patients exhibited significant abundance over that in the saliva of healthy controls using 16S rDNA sequencing. Moreover, it was also reported that the salivary microbial profile could reflect the periodontopathogens in subgingival plaque sample [11,12,13]. However, there are only a limited number of documentations of the correlations of different bacterial sampling methods in detection of bacteria that are correlated with pathologic periodontal conditions.

Various methods have been adopted for the detection of bacteria. Cultivation technique, microscopic evaluation, immunological methods, and DNA hybridization-based methods have elucidated different microbiological characteristics in periodontitis patients. However, the majority of the detection methods mentioned above are semi-quantitative, expensive, often time-consuming and labor-intensive. Currently, multiplex real-time polymerase chain reaction (PCR) received attention for its ability to detect several target DNA sequences simultaneously, and high-throughput quantification with less sample and input material. Nevertheless, there are only limited numbers of documentations available that adopted multiplex real-time PCR for the identification and quantification of microbiological profiles. For example, Estrela and colleagues [14] reported a prevalence of bacterial species in samples from different intraoral sites of periodontitis patients using multiplex real-time PCR, but there were no data on quantity of different bacterial species. More recently, a research performed by Lochman [15] presented the quantification of cariogenic and periodontopathic bacteria from 30 Czech children who had severe early childhood caries and gingivitis, without including periodontitis of adult subjects.

There are limited numbers of research studies that evaluated the quantitative assessment of bacterial profile of periodontitis patients using multiplex real-time PCR. The aim of the present study was to quantitatively compare the bacterial profile of patients with different severity of periodontal disease using samples from saliva and the subgingival area. An additional purpose was to evaluate the correlation between the salivary and subgingival bacterial profile using multiplex real-time PCR, ultimately evaluating the microbiological diagnostic performance of saliva compared to other sampling methods in patients with periodontal diseases.

## 2. Materials and Methods

### 2.1. Ethical Approval and Study Population

This study was approved by Institutional Review Board of Seoul National University Dental Hospital (Code: CRI18002, 19 October 2017) and was conducted with strict observance of the Declaration of Helsinki. Sufficient information on the clinical study was given to all participants and written informed consents were obtained at their own free will before enrollment in the study.

The inclusion criteria of study population were presented as follows: age of 20 to 69 years old who visited Seoul National University Dental Hospital from December 2018 to March 2020, having at least 20 natural teeth, and absence of any systemic disease. Subjects were excluded from the study if they had antibiotic therapy within 2 weeks from the study date, if they had periodontal treatment within 6 months from the study date, and if they were under orthodontic treatment.

### 2.2. Clinical Examination and Study Group Assignment

After enrollment in the study, all participants underwent full mouth recording of various periodontal parameters, including probing pocket depth (PPD), and clinical attachment loss (CAL), bleeding on probing (BOP), plaque index (PI), and gingival index (GI), which were registered at six sites (mesio-facial, mid-facial, disto-facial, mesio-lingual, mid-lingual, and disto-lingual) of all teeth except third molars and dental implants. Panoramic x-ray was taken to evaluate alveolar bone loss and to screen any other clinically significant pathology.

Based on the result of clinical examination and radiographic evaluation, all participants were assigned to one of the three study groups. The classification of different study groups was originated and modified from the case definition introduced by the US Centers for Disease Control and Prevention and the American Academy of Periodontology [16].

- Group 1 (Severe periodontitis, SP): presence of 2 or more interproximal sites with ≥6 mm of CAL and 1 or more interproximal site(s) with ≥5 mm of PPD
- Group 2 (Moderate periodontitis, MP): 2 or more interproximal sites with ≥4 mm of CAL or 2 or more interproximal sites with ≥5 mm of PPD
- Group 3 (Gingivitis/Mild periodontitis, G/M): subjects who are not assigned to Group 1 or 2.

### 2.3. Microbial Sampling

All subjects were refrained from eating or drinking anything and from toothbrushing at least 3 hours before sample collection. For salivary sample collection, 12 mL of mouthwash solution provided by analytical company (Periogen, Gyeonggi-do, Korea) was given to all subjects to rinse their mouth for 30 s. After rinsing, the mouthwash solution was spitted into a sample collection tube and the cap of the tube was closed tightly. For subgingival microbial sampling, two teeth from every individual who presented the deepest PPD and similar periodontitis lesions were selected. Before subgingival sampling, supragingival dental biofilm was gently removed and all sampling sites were isolated from saliva. Three sterile ISO #35 paperpoints were inserted into three of six periodontal pocket sites of one representative tooth for 30 s. One sterile gingival retraction cord of 10 mm in length was inserted into the periodontal pocket of the other representative tooth for 30 s. After retrieval from each periodontal pocket, both paperpoint and gingival retraction cord samples were immediately transferred to an EP-tube containing 1 mL of the mouthwash solution mentioned above. All samples were stored in a refrigerator at 4 °C before DNA extraction.

### 2.4. DNA Extraction and Multiplex Real-Time PCR

Bacterial DNA was extracted using Exgene Cell SV mini kit (GeneAll, Seoul, Korea). The extracted DNA samples were stored at −20 °C before any further analyses. The samples were analyzed using a real-time PCR kit (GeneAll, Seoul, Korea) to detect the following periodontopathic bacteria: *Aggregatibacter actinomycetemcomitans* (*Aa*), *Porphyromonas gingivalis* (*Pg*), *Tannerella forsythia* (*Tf*), *Treponema denticola* (*Td*), *Prevotella intermedia* (*Pi*), *Fusobacterium nucleatum* (*Fn*), *Parvimonas micra* (*Pm*), *Campylobacter rectus* (*Cr*), *Eiknella corrodens* (*Ec*), *Prevotella nigrescens* (*Pn*), *Eubacterium nodatum* (*En*). Each bacterial DNA sample was amplified by the specific primer that targets functional gene (e.g., rpgB, waaA, gtf) of each species.

For the samples to be analyzed in the Hot-start Taq DNA polymerase assay, all samples were processed in 20 µL reaction mixture, containing 2 µL of extracted DNA solution, periodontal pathogen-specific primers (Periogen, Gyeonggi-do, Korea), and PCR reaction buffer. PCR analyses were conducted with ABI 7500 Fast Real-Time PCR System (Life Technologies, Carlsbad, CA, USA). After initial denaturation at 95 °C for 15 min, 40 cycles of amplification were programmed, each amplification being composed of 95 °C for 30 s, 55 °C for 30 s, and 72 °C for 30 s.

Standard curves were constructed with known amounts of bacterial DNA, plotting the relationship between cycle threshold (Ct) values and the numbers of bacterial DNA copies. The obtained Ct value of each bacterial sample was converted into the DNA copy numbers, which were used in the quantitative comparison procedures.

### 2.5. Statistical Analysis

All statistical analyses were performed using SPSS 25.0 (IBM Corp., Armonk, NY, USA). All data were checked on their normality with the Shapiro–Wilk test. If the data set could be assumed to follow Gaussian distribution, one-way ANOVA and Dunnet T3 test for post hoc analysis was performed. Nonparametric data sets were compared with the Kruskal–Wallis H test, and Bonferroni correction was adopted for multiple comparison. Prevalence of certain bacteria in each sample was compared with the Pearson’s Chi-square test. Correlations between data sets were analyzed with Spearman’s rank correlation. *p* values < 0.05 were set to indicate statistical significance. All graphs were plotted using GraphPad Prism 7 (GraphPad Software, San Diego, CA, USA).

## 3. Results

### 3.1. Demographics and Clinical Data

115 subjects were volunteered to be enrolled in this study, but one subject was excluded due to ongoing orthodontic treatment; 114 participants were divided into 3 groups that were previously described, 38 subjects comprising each study group.

Demographic and clinical data are described in detail in Table 1. Data that follow Gaussian distribution were described with mean ± standard deviation, and nonparametric data were expressed with median and interquartile range in parentheses. There were statistically significant differences on age (SP group: 58.5 (51.5–61.0), MP group: 42.5 (37.0–55.0), G/M group: 27.5 (23.0–37.25)), PPD (SP group: 2.894 ± 0.378 mm, MP group: 2.490 ± 0.265 mm, G/M: 2.274 ± 0.167 mm) and CAL (SP group: 3.382 (3.015–3.619), MP: 2.651 (2.453–2.835), G/M: 2.266 (2.196–2.393)) between all study groups. GI was significantly different between MP group (0.143 (0.038–0.594)) and G/M group (0.417 (0.278–0.656)). Sex distribution, BOP%, and PI were not significantly different between all study groups.

### 3.2. Prevalence of Target Bacteria

Prevalence of target bacteria is presented according to the different sampling strategies, that is, paperpoint (Table 2), gingival retraction cord (Table 3), and mouthwash (Table 4), respectively. *Fn* were detected with high frequencies among all samples, regardless of the sampling methods. In paperpoint sample, six bacteria (*Pg, Tf, Td, Pm, Cr*, and *En*) presented significantly heterogenous prevalence between study groups. Similarly, six bacteria (*Pg, Tf, Td, Pi, Cr*, and *En*) showed unequal distribution of prevalence between study groups. However, in mouthwash samples, significantly different prevalence were detected only from two bacteria (*Pg, Tf*).

### 3.3. Quantitative Profiles of Target Bacteria

**Figure 1:**
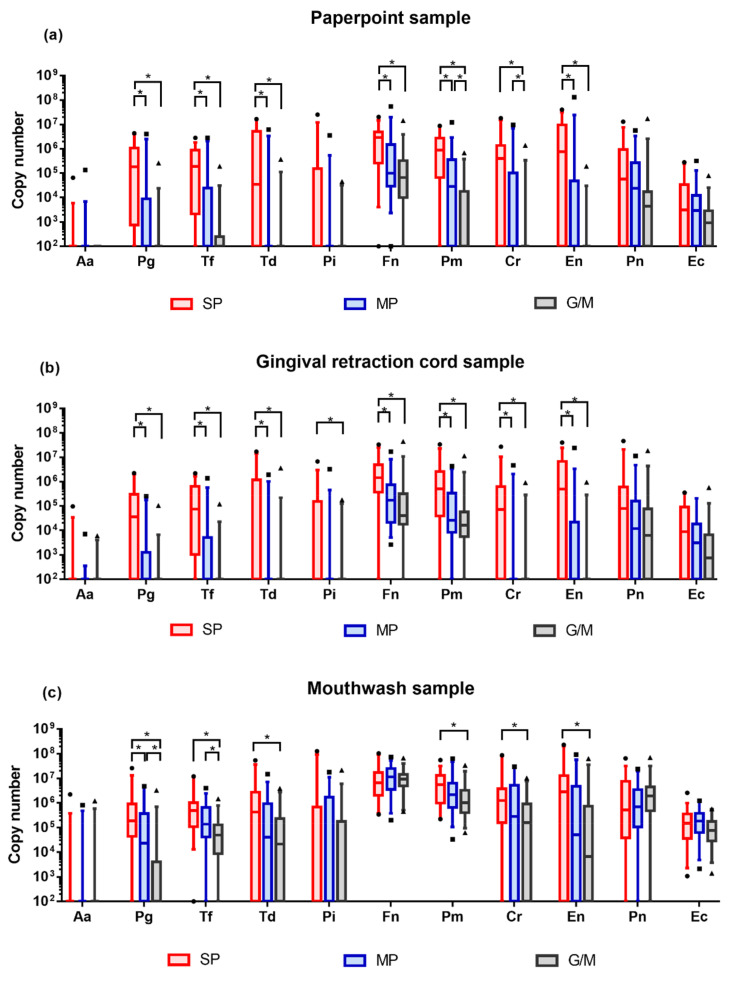
Quantitative profiles of target bacteria. DNA copy numbers are reported on a log_10_ scale. DNA copy numbers are plotted with box and whisker plot, indicating interquartile range and 5–95 percentile, respectively. All comparisons were conducted with Kruskal–Wallis H test and Bonferroni correction. Adjusted *p*-value = 0.0167. (**a**) Bacterial profile in the paperpoint samples; (**b**) Bacterial profile in the gingival retraction cord samples; (**c**) Bacterial profile in the mouthwash samples. SP: severe periodontitis, MP: moderate periodontitis, G/M: gingivitis/mild periodontitis.

Bacterial DNA copy numbers were converted from Ct values that were obtained from multiplex real-time PCR analyzer. Different profiles were depicted to compare the difference between study groups in the same sampling method (Figure 1, Table A1, Table A2 and Table A3).

Obtained bacterial DNA exhibited highly diverse quantities. The majority of the target bacteria showed increasing tendency in their amount as the severity of periodontal disease deteriorated. In paperpoint samples, DNA copy numbers of 7 bacteria (*Pg, Tf, Td, Fn, Pm, Cr,* and *En*) presented significant differences between study groups. In gingival retraction cord samples, similar profiles were observed, indicating significant differences in 8 bacterial species (*Pg, Tf, Td, Pi, Fn, Pm, Cr,* and *En*). In mouthwash samples, 6 bacterial (*Pg, Tf, Td, Pm, Cr,* and *En*) exhibited statistical difference in their DNA copy numbers. Regardless of the sampling methods, the majority of the statistical differences were observed between severe periodontitis and moderate periodontitis, and between severe periodontitis and gingivitis/mild periodontitis.

### 3.4. Correlations between Sampling Methods

The DNA copy number in mouthwash samples was correlated with that in the paperpoint sample and that in the retraction cord sample in each study group using Spearman’s correlation analysis. Spearman’s correlation coefficients (*ρ*) of each bacterial species in SP, MP, and G/M groups were summarized below (Table 5).

Both subgingival sampling methods presented similar correlations with the mouthwash sampling method in broad outlines. In the G/M group, statistically significant correlation coefficients fall into the range of 0.3–0.5, which indicates fair correlation [17]. However, in the SP and MP groups, compared to the G/M group, many correlation coefficients that present statistical significance belong in the range of 0.4–0.8, suggesting fair to moderately strong correlation. The results may imply that mouthwash samples were more strongly correlated with site-specific subgingival samples as the severity of periodontal disease increases.

### 3.5. Correlations between Periodontal Disease Severity and Various Parameters

The severity of periodontal disease was correlated with various clinical parameters and bacterial parameters using Spearman’s correlation analysis (Table 6). Bacterial parameters consisted of DNA counts of each bacterial species in mouthwash samples, and the feasibility of salivary diagnostics of periodontitis was examined. The severity of periodontal disease presented statistically significant correlation with mean PPD, mean CAL (*ρ* = 0.664, 0.792, respectively). In bacterial parameters, *Pg, Tf, Td, Pm, Cr* and *En* in mouthwash samples were significantly correlated with the severity of periodontal disease (*ρ* = 0.530, 0.438, 0.209, 0.276, 0.283, 0.311, respectively). However, only *Pg* and *Tf* in mouthwash exhibited fair correlation with the severity of periodontal disease.

## 4. Discussion

In the present study, 11 target periodontopathic bacteria, which were mainly ‘Red complex’ and ‘Orange complex’, were selected and tested for their feasibility of diagnostic application, as bacteria were closely related to the pathogenesis of periodontitis. Our data demonstrated that the majority of target bacteria exhibited increased counts both in mouthwash and in subgingival samples as the severity of periodontal disease increased. Regardless of the sampling methods, *Pg, Tf, Td, Pm, Cr*, and *En* presented significant differences between study groups. This result is similar to that of a previous study presenting higher *Pg* and *Tf* level in periodontitis patients [18].

From our data, the prevalence and quantity of periodontopathic bacteria in subgingival samples tend to increase as the disease severity increases. Notably, *Fn* was detected with high frequency and quantity from all samples. This result is in line with previous study demonstrating that *Fn* was the most abundant in both conditions of healthy and periodontitis [19]. *Fn* is known as a bridging species linking early colonizers on tooth surface and late-colonizing pathogens, such as ‘Red complex’ [20,21]. Significant increase of *Fn* in severe periodontitis was observed in subgingival samples, but the differences in amount between study groups were insignificant in mouthwash samples. This may be explained by the ubiquity of *Fn* in the oral cavity, as it is capable of binding to oral epithelial cells [22]. Despite the increase of *Fn* in the subgingival area and its flush-out into oral cavity, it may be masked by previously populated *Fn* in oral cavity.

Various sampling techniques were adopted for the analysis of subgingival plaque. Curette [6,23] or paperpoint [24,25] was the most frequently utilized in the subgingival sampling procedure. Previous studies demonstrated the relationship between the subgingival sampling methods. It was reported that although curette sampling could represent higher total bacterial counts than paperpoint sampling, the plaque composition of target bacteria was similar for both sampling methods, suggesting that both sampling techniques can be used in microbial assessment [26]. Belibasakis and colleagues [27] reported similar profiles of ‘Red complex’ both from paperpoint and from curette samples. In another study, ligature that induced experimental periodontitis and paperpoint subsequently inserted in the same sites after ligature removal were compared with checkerboard DNA-DNA hybridization [28]. Considering that similar bacterial profiles between the ligature and paperpoint, it is suggested that a ligature on a tooth may also be used as a sampling method. In the present study, the gingival retraction cord, which resembles the ligature used in the animal experiment, was utilized for subgingival sampling. Gingival retraction cord was assumed to be more reproducible than paperpoint, because it would cover a larger surface of subgingival area, resulting in effective reflection of subgingival microbiota. Moreover, paperpoint would be deformed or folded after absorption of gingival crevicular fluid. This might hinder the insertion of paperpoint deep into the periodontal pocket, and gingival retraction cord would be less technique-sensitive than paperpoint [29]. However, as the result of the present study exhibited, there was no significant difference in the amount of target bacterial counts between two subgingival sampling methods. This implies that the gingival retraction cord may also be utilized in the subgingival biofilm sampling procedure.

Saliva was featured as a promising source of periodontopathic bacteria because of its easiness and non-invasiveness of sampling. Instead of saliva, mouthwash sampling was suggested for the detection of bacterial DNA since it was more straightforward and faster than saliva collection [17]. Since mouthwash contains several antiseptics and alcohol, it can prevent bacterial growth during the entire sampling procedure and cooling step for storage. This was supported by one study reporting that overall bacterial composition was not significantly different between mouthwash sample and saliva sample [30]. Another study demonstrated that the utilization of commercially available mouthwash yielded a significant amount of human genomic DNA from buccal cells with high quality [31]. Taken together, it could be suggested that the mouthwash sampling would not significantly degrade bacterial DNA in the mouthwash solution. Hence, mouthwash sampling may be considered an alternative to the saliva sampling.

In our data, *Pg, Tf, Td, Pm, Cr* and *En* in the mouthwash presented a significant difference between study groups. Among them, *Pg* only presented a significant difference between all study groups. In addition, *Pg* in mouthwash was correlated with the severity of periodontal disease, exhibiting greatest correlation coefficient of 0.530. This indicates that the *Pg* in mouthwash may serve as a bacterial biomarker for periodontitis. This is consistent with previous studies demonstrating different salivary *Pg* profiles in periodontitis patients and healthy subjects [32,33]. More recently, salivary microbiota was analyzed with sequencing-based method, and also demonstrated that the amount of salivary *Pg* was more prominent in periodontitis patients than that in healthy subjects [34,35]. Based on these results, it may be suggested that *Pg* in saliva or mouthwash has potential to be utilized as a diagnostic marker of periodontitis.

The bacterial profile of mouthwash samples was correlated with that of subgingival samples in this study. The majority of the correlation coefficients of target bacteria presented significant correlation between mouthwash and subgingival samplings. Notably, as the severity of periodontal disease increased (from gingivitis/mild periodontitis to severe periodontitis), the correlations became stronger. This can be explained by the combinatorial effect of bacterial proliferation and the flow of gingival crevicular fluid. The subgingival periodontopathic bacteria increase in deep periodontal pocket as the severity of periodontal disease deteriorates [36]. In addition, the flow of gingival crevicular fluid increases and its flushing action is reinforced in the inflammatory conditions [37]. As a result, the increased bacterial DNA is washed out to oral cavity more abundantly, and finally is detected with mouthwash sampling.

The tendency of enhanced correlation between mouthwash sample and subgingival sample in the severe periodontitis group was demonstrated in the present study. In addition, the quantitative profile did not significantly discriminate moderate periodontitis and gingivitis/mild periodontitis in the majority of targeted bacterial species. These may imply that the microbiological diagnosis using mouthwash sampling may be more suitable for diagnostic application in severe periodontitis, rather than mild or moderate periodontitis. This may represent the difficulty of early diagnosis of periodontitis using microbiological assessment. Although microbiological examination with multiplex real-time PCR may not be suitable for early diagnosis of periodontitis, it still has some values in the management of periodontitis. First, it can be adopted in the decision-making procedure of antibiotics use. Aggressive periodontitis or periodontitis with refractory characteristics which did not respond to mechanical debridement well can be managed with the administration of antibiotics. Second, it may be utilized in patient monitoring for the recurrence of periodontitis. Since it is reported that an increase of some periodontopathic bacteria over a certain threshold in regular recall check-up was correlated with 2.5 times greater risk of disease recurrence [38], microbiological test may be helpful in the maintenance care of periodontitis-susceptible patients.

## 5. Conclusions

Using multiplex real-time PCR, it was demonstrated that major periodontopathic bacteria presented different bacterial profiles and prevalence among the study groups with different severity of periodontal disease. Regardless of the sampling methods, 6 bacterial species (*Pg, Tf, Td, Pm, Cr*, and *En*) presented significant differences between different periodontal disease severity. Many bacterial species tested in mouthwash exhibited significant correlation with that in subgingival samples, demonstrating the possibility that bacteria in mouthwash can reflect that in the subgingival area. This may imply that bacterial count in mouthwash as a potential biomarker for the diagnosis of periodontitis. However, considering the correlation was enhanced as the severity of periodontitis deteriorates, it might be difficult to diagnose periodontitis in the early stage of tissue destruction using microbiological assessment. In future, longitudinal study of monitoring the periodontal health in relation to the periodontopathic bacteria may be performed to evaluate the efficacy of microbiological examination in the diagnosis and the management of periodontitis. In addition, the discovery of biomarkers that can report early diagnosis of periodontitis may be desired, and bacterial biomarkers might be concomitantly utilized with the newly developed biomarkers, such as interleukin-1, matrix metalloproteinase-8 and other protein biomarkers, in the diagnosis of the periodontitis.

## Figures and Tables

**Table 1:** Demographic and clinical data of study groups.

	SP Group	MP Group	G/M Group	*p*
Participants number	38	38	38	
Age (years) ^1^	58.5 (51.5–61.0)	42.5 (37.0–55.0)	27.5 (23.0–37.25)	<0.001
Female: Male	22: 16	22: 16	25: 13	0.719
PPD (mm) ^2^	2.894 (±0.378)	2.490 (±0.265)	2.274 (±0.167)	<0.001
CAL (mm) ^1^	3.382 (3.015–3.619)	2.651 (2.453–2.835)	2.266 (2.196–2.393)	<0.001
BOP%	53.910 (±26.826)	47.126 (±20.334)	49.015 (±20.190)	0.394
PI	0.275 (0.194–0.664)	0.211 (0.122–0.451)	0.280 (0.176–0.414)	0.196
GI ^3^	0.295 (0.086–0.436)	0.143 (0.038–0.594)	0.417 (0.278–0.656)	0.009

^1^ Significant difference between all study groups. Kruskal–Wallis H test and Bonferroni correction. ^2^ Significant difference between all study groups. One-way ANOVA and Dunnet T3 test. ^3^ Significant difference between MP group and G/M group. Kruskal–Wallis H test and Bonferroni correction. SP: severe periodontitis, MP: moderate periodontitis, G/M: gingivitis/mild periodontitis, PPD: probing pocket depth, CAL: clinical attachment loss, BOP: bleeding on probing, PI: plaque index, GI: gingival index.

**Table 2:** Prevalence of target bacteria in paperpoint samples of different study groups.

	*Aa*	*Pg*	*Tf*	*Td*	*Pi*	*Fn*	*Pm*	*Cr*	*En*	*Pn*	*Ec*
SP (%)	5.26	76.32	78.95	52.63	28.95	97.37	86.84	55.26	63.16	60.53	65.79
MP (%)	2.63	36.84	44.74	21.05	18.42	97.37	65.79	39.47	26.32	71.05	60.53
G/M (%)	0	15.79	23.68	15.79	10.53	94.74	47.37	7.89	7.89	52.63	55.26
*p* ^1^	0.358	<0.001	<0.001	0.004	0.124	0.772	0.007	<0.001	<0.001	0.317	0.644

SP: severe periodontitis, MP: moderate periodontitis, G/M: gingivitis/mild periodontitis. ^1^ *p*-values were calculated by Pearson’s Chi-square test.

**Table 3:** Prevalence of target bacteria in gingival retraction cord samples of different study groups.

	*Aa*	*Pg*	*Tf*	*Td*	*Pi*	*Fn*	*Pm*	*Cr*	*En*	*Pn*	*Ec*
SP (%)	10.53	65.79	76.32	39.47	21.05	94.74	86.84	52.63	65.79	60.53	68.42
MP (%)	2.63	26.32	42.11	15.79	13.16	100	78.95	21.05	26.32	57.89	65.79
G/M (%)	5.26	10.53	18.42	5.26	7.89	92.11	76.32	10.53	7.89	63.16	55.26
*p* ^1^	0.345	<0.001	<0.001	0.001	0.037	0.231	0.481	<0.001	<0.001	0.896	0.712

SP: severe periodontitis, MP: moderate periodontitis, G/M: gingivitis/mild periodontitis. ^1^ *p*-values were calculated by Pearson’s Chi-square test.

**Table 4:** Prevalence of target bacteria in mouthwash samples of different study groups.

	*Aa*	*Pg*	*Tf*	*Td*	*Pi*	*Fn*	*Pm*	*Cr*	*En*	*Pn*	*Ec*
SP (%)	13.16	89.47	97.37	63.16	36.84	100	100	81.58	73.68	84.21	100
MP (%)	10.53	63.16	94.74	55.26	47.37	100	100	65.79	57.89	84.21	100
G/M (%)	5.26	23.68	81.58	57.89	31.58	100	100	57.89	50.00	89.47	100
*p* ^1^	0.494	<0.001	0.033	0.867	0.355	-	-	0.077	0.099	0.748	-

SP: severe periodontitis, MP: moderate periodontitis, G/M: gingivitis/mild periodontitis. ^1^ *p*-values were calculated by Pearson’s Chi-square test.

**Table 5:** Correlations between different sampling methods for the detection of each bacterial species in different study groups. Mouthwash samples (M) were correlated with paperpoint samples (P) and gingival retraction cord samples (C).

		*Aa*	*Pg*	*Tf*	*Td*	*Pi*	*Fn*	*Pm*	*Cr*	*En*	*Pn*	*Ec*
SP	*ρ_P-M_*	0.284	0.640	0.591	0.823	0.606	0.449	0.581	0.665	0.585	0.555	0.293
	*p*	0.084	<0.001	<0.001	<0.001	<0.001	0.005	<0.001	<0.001	<0.001	<0.001	0.075
	*ρ_C-M_*	0.656	0.793	0.705	0.753	0.534	0.444	0.661	0.807	0.734	0.480	0.238
	*p*	<0.001	<0.001	<0.001	<0.001	0.001	0.005	<0.001	<0.001	<0.001	0.002	0.149
MP	*ρ_P-M_*	0.464	0.743	0.513	0.552	0.438	0.306	0.415	0.556	0.660	0.361	0.256
	*p*	0.003	<0.001	0.001	<0.001	0.006	0.062	0.010	<0.001	<0.001	0.026	0.120
	*ρ_C-M_*	0.492	0.506	0.723	0.631	0.576	0.496	0.508	0.625	0.742	0.617	0.289
	*p*	0.002	0.001	<0.001	<0.001	<0.001	0.002	0.001	<0.001	<0.001	<0.001	0.078
G/M	*ρ_P-M_*	-	0.463	0.424	0.434	0.380	0.431	0.242	0.206	0.418	0.063	0.496
	*p*	-	0.003	0.008	0.006	0.019	0.007	0.143	0.215	0.009	0.705	0.002
	*ρ_C-M_*	0.500	0.237	0.442	0.351	0.316	0.412	0.372	0.308	0.326	0.334	0.182
	*p*	0.001	0.152	0.006	0.031	0.053	0.010	0.021	0.060	0.046	0.041	0.274

SP: severe periodontitis, MP: moderate periodontitis, G/M: gingivitis/mild periodontitis. *ρ_P-M_*: Spearman’s correlation coefficient between paperpoint sample and mouthwash sample, *ρ_C-M_*: Spearman’s correlation coefficient between gingival retraction cord sample and mouthwash sample.

**Table 6:** Correlations between the severity of periodontal disease and clinical parameters and bacterial parameters. All bacterial parameters indicate the DNA counts of each bacterial species in the mouthwash samples.

Clinical parameters		**PPD**		**CAL**		**BOP%**		**PI**		**GI**		
	*ρ*	0.664		0.792		0.089		0.048		−0.181		
	*p*	<0.001		<0.001		0.345		0.613		0.054		
Bacterial parameters		***Aa***	***Pg***	***Tf***	***Td***	***Pi***	***Fn***	***Pm***	***Cr***	***En***	***Pn***	***Ec***
	*ρ*	0.100	0.530	0.438	0.209	0.076	−0.104	0.276	0.283	0.311	−0.091	0.147
	*p*	0.290	<0.001	<0.001	0.026	0.419	0.272	0.003	0.002	0.001	0.337	0.118

SP: severe periodontitis, MP: moderate periodontitis, G/M: gingivitis/mild periodontitis. ρ: Spearman’s correlation coefficient between the severity of periodontal disease and various parameters.

## Appendix A

**Table A1:** Bacterial DNA counts of target bacteria in paperpoint samples of different study groups. Median values and interquartile ranges were summarized in the table.

	*Aa*	*Pg* ^1,2^	*Tf* ^1,2^	*Td* ^1,2^	*Pi*	*Fn* ^1,2^	*Pm* ^1,2,3^	*Cr* ^2,3^	*En* ^1,2^	*Pn*	*Ec*
SP	0	183,323.5	189,716.5	34,937	0	2,878,321.5	873,304.5	393,711.5	751,512	56,823.5	3,134.5
	(0–0)	(748.5–1,074,205)	(2,167.25–883,077.5)	(0–5,165,656.25)	(0–153,263)	(255,422.25–4,941,155.5)	(65,136.75–2,773,312)	(0–1,379,595.75)	(0–9,643,960.25)	(0–926,327.5)	(0–34,212.25)
MP	0	0	0	0	0	99,739	28,384	0	0	23,634	2,933.5
	(0–0)	(0–8,918.5)	(0–24,334.75)	(0–0)	(0–0)	(29,533.5–1,502,432.25)	(0–352,695.5)	(0–100,546.25)	(0–48,412.75)	(0–266,390.5)	(0–12,331.5)
G/M	0	0	0	0	0	66,850	0	0	0	4,481	934.5
	(0–0)	(0–0)	(0–253)	(0–0)	(0–0)	(10,112–325,644.5)	(0–17,599.75)	(0–0)	(0–0)	(0–17,236)	(0–2,831.75)

^1^ Significant difference between SP group and MP group, Kruskal–Wallis H test and Bonferroni correction, adjusted *p* value = 0.0167. ^2^ Significant difference between SP group and G/M group, Kruskal–Wallis H test and Bonferroni correction, adjusted *p* value = 0.0167. ^3^ Significant difference between MP and G/M group, Kruskal–Wallis H test and Bonferroni correction, adjusted *p* value = 0.0167. SP: severe periodontitis, MP: moderate periodontitis, G/M: gingivitis/mild periodontitis.

**Table A2:** Bacterial DNA counts of target bacteria in gingival retraction cord samples of different study groups. Median values and interquartile ranges were summarized in the table.

	*Aa*	*Pg* ^1,2^	*Tf* ^1,2^	*Td* ^1,2^	*Pi* ^2^	*Fn* ^1,2^	*Pm* ^1,2^	*Cr* ^1,2^	*En* ^1,2^	*Pn*	*Ec*
SP	0	35,508.5	74,978.5	0	0	1,455,404.5	503,886	71,779	500,946	79,486	8,940.5
	(0–0)	(0–307,175)	(1,042.5–647,178.25)	(0–1,194,157.25)	(0–152,042.5)	(367,937.25–4,884,594.25)	(39,334–2,572,092.75)	(0–631,647.75)	(0–6,867,778)	(0–606,872.75)	(0–90,470.5)
MP	0	0	0	0	0	170,745.5	26,252	0	0	12,051.5	3,093
	(0–0)	(0–1,265.5)	(0–5,182)	(0–0)	(0–0)	(20,939.25–737,556)	(8,414.5–342,992)	(0–0)	(0–22,114)	(0–157,424.25)	(0–18,200.75)
G/M	0	0	0	0	0	40,459.5	16,467.5	0	0	6,291	748
	(0–0)	(0–0)	(0–0)	(0–0)	(0–0)	(18,085.25–325,196.75)	(5,637–57,035)	(0–0)	(0–0)	(0–77,090.25)	(0–6,699.25)

^1^ Significant difference between SP group and MP group, Kruskal–Wallis H test and Bonferroni correction, adjusted *p* value = 0.0167. ^2^ Significant difference between SP group and G/M group, Kruskal–Wallis H test and Bonferroni correction, adjusted *p* value = 0.0167. ^3^ Significant difference between MP and G/M group, Kruskal–Wallis H test and Bonferroni correction, adjusted *p* value = 0.0167. SP: severe periodontitis, MP: moderate periodontitis, G/M: gingivitis/mild periodontitis.

**Table A3:** Bacterial DNA counts of target bacteria in mouthwash samples of different study groups. Median values and interquartile ranges were summarized in the table.

	*Aa*	*Pg* ^1,2,3^	*Tf* ^2,3^	*Td* ^2^	*Pi*	*Fn*	*Pm* ^2^	*Cr ^2^*	*En ^2^*	*Pn*	*Ec*
SP	0	188,897.5	497,370.5	433,115.5	0	6,554,428.5	5,539,908.5	1,238,897.5	2,864,722.5	530,676	151,356.5
	(0–0)	(43,845.75–916,819.75)	(107,559.5–1,019,966.25)	(0–2,716,230.5)	(0–690,047.75)	(2,063,203.75–17,091,004.75)	(986,647.75–13,044,378.25)	(157,592.25–3,809,185.75)	(0–12,768,964.5)	(37,772–7,668,221.5)	(36,227.5–356,423.75)
MP	0	23,046	138,542.5	40,535	0	11,526,033	2,169,859.5	285,393.5	50,889	699,288.5	186,822.5
	(0–0)	(0–368,919.5)	(41,235.75–656,630.5)	(0–946,794)	(0–1,736,808.5)	(3,614,555.25–25,268,039.5)	(654,273.25–6,432,139)	(0–5,267,433.25)	(0–4,742,616.25)	(105,581–3,472,560.25)	(62,743.75–380,438)
G/M	0	0	49,497.5	21,070.5	0	9,337,878	1,039,920	157,564.5	6,667.5	1,963,360	75,491
	(0–0)	(0–4,036)	(8,594.75–128,008)	(0–230,996.25)	(0–180,066.5)	(4,918,633.25–14,351,886.25)	(406,851.5–3,298,036)	(0–900,288)	(0–749,423.25)	(476,101–4,538,996.75)	(28,721.5–181,810.25)

^1^ Significant difference between SP group and MP group, Kruskal–Wallis H test and Bonferroni correction, adjusted *p* value = 0.0167. ^2^ Significant difference between SP group and G/M group, Kruskal–Wallis H test and Bonferroni correction, adjusted *p* value = 0.0167. ^3^ Significant difference between MP and G/M group, Kruskal–Wallis H test and Bonferroni correction, adjusted *p* value = 0.0167. SP: severe periodontitis, MP: moderate periodontitis, G/M: gingivitis/mild periodontitis.
